# Positive and Negative Experiences of Living in COVID-19 Pandemic: Analysis of Italian Adolescents’ Narratives

**DOI:** 10.3389/fpsyg.2020.599531

**Published:** 2020-11-19

**Authors:** Chiara Fioretti, Benedetta Emanuela Palladino, Annalaura Nocentini, Ersilia Menesini

**Affiliations:** Department of Education, Languages, Intercultures, Literatures and Psychology, University of Florence, Firenze, Italy

**Keywords:** adolescence, COVID-19, narratives, identity, qualitative research

## Abstract

**Introduction:**

Despite a growing interest in the field, scarce narrative studies have delved into adolescents’ psychological experiences related to global emergencies caused by infective diseases. The present study aims to investigate adolescents’ narratives on positive and negative experiences related to COVID-19.

**Methods:**

Italian adolescents, 2,758 (females = 74.8%, mean age = 16.64, *SD* = 1.43), completed two narrative tasks on their most negative and positive experiences during the COVID-19 emergency. Data were analyzed by modeling an analysis of emergent themes.

**Results:**

“Staying home as a limitation of autonomy,” “School as an educational, not relational environment,” the impact of a “new life routine,” and experiencing “anguish and loss” are the four emergent themes for negative experiences. As for positive experiences, the four themes were “Being part of an extraordinary experience,” “Discovering oneself,” “Re-discovering family,” and “Sharing life at a distance.”

**Conclusion:**

Authors discuss the impact of COVID-19 on adolescents’ developmental tasks, such as identity processes and autonomy acquisition.

## Introduction

After the first case of COVID-19 in Italy was discovered on the 21st of February, schools and universities were shut down on March 5. On the March 9, the government declared lockdown status in order to hinder the spread of the virus. In order to reduce contagion, citizens were required to stay home except for emergencies and primary needs. Over 8 million children and adolescents stopped their social and educational activities, which were reorganized online. On April 5, the last day of data collection for the present study, out of a global number of 1,133,758 (Johns Hopkins Coronavirus Resource Center, 2020), 128,948 people had been infected by COVID-19 in Italy, of which 15,887 (about the 12.3%) had died (Italian Ministry of Health, 2020).

The COVID-19 pandemic is a public health emergency that poses questions and dilemmas regarding the psychological well-being of people at varying levels.

Currently, several studies have been conducted on how the general population experiences emergencies related to pandemic infectious diseases. Some authors (Yeung and Fung, 2007; Dodgson et al., 2010; Peng et al., 2010; Main et al., 2011; Van Bortel et al., 2016), in analyzing the impact of infectious diseases such as SARS or Ebola, report experiences such as fear and anxiety for themselves and their families, separation anxieties, impotence, depression, as well as anger and frustration. In the case of COVID-19, scholars have highlighted several psychological effects of the pandemic on adult samples in China (Qiu et al., 2020; Wang et al., 2020a,b) and in Italy (Rossi et al., 2020), and found psychological symptoms related to posttraumatic stress disorder. In a recent review, anxiety, depression, psychological stress, and poor sleep have been reported to be the main psychological outcomes of living with the COVID-19 emergency (Rajkumar, 2020).

Considering children and adolescents, several studies have specifically explored psychological experiences related to the global emergency and lockdown experience of COVID-19 (Lee, 2020), but evidence from autobiographical narratives are lacking. Qiu et al. (2020) compared different Chinese aged populations and found lower levels of psychological distress in people under 18. Similarly, Xie et al. (2020) found symptoms of anxiety (18.9%) and depression (22.6%) in primary school children in China.

As for US adolescents, evidence suggests that social trust and greater attitudes toward the severity of COVID-19 are related with more adolescents’ monitoring risk behaviors, performing social distancing, and disinfecting properly. Motivation to perform social distancing is also associated with symptoms of anxiety and depression (Oosterhoff et al., 2020).

A study on Canadian adolescents’ well-being and psychiatric symptoms highlighted that depression and feelings of loneliness are related with great time spent on social media, while family time, physical activity, and schoolwork play a protective role for depression (Ellis et al., 2020). Similarly, in a recent review of adolescents’ experience of lockdown for COVID-19, Guessoum et al. (2020) discuss the relation between the current pandemic and adolescents’ posttraumatic stress, depressive, and anxiety disorders, as well as grief-related symptoms. Furthermore, they found that data on adolescent mental health are still scarce and need to be empowered.

Adolescence is connected to certain developmental tasks (Havighurst, 1948) related, among others, to defining one’s own personal identity (Kroger and Marcia, 2011) and developing one’s autonomy by redefining family ties and building bonds with peers (Alonso-Stuyck et al., 2018).

Considering identity changes, adolescence is characterized by a developmental crisis between the definition of a personal identity and a status of confusion of roles (Erikson, 1968). Adolescents’ ego growth is linked to the separation from childhood identifications in order to allow an individual identity status to emerge. This gradual process is connected with four different styles of identity definition concerning vocational, ideological, and sexual issues (Kroger et al., 2010): identity achievements, moratorium, foreclosure, and diffusion. Overall, the identity process may develop from a period of diffusion, not connected to significant identifications, or with foreclosure, in which identifications are still related to significant childhood figures. The opportunity to explore new relationships with peers and other developmental environments often stresses a time of identity moratorium where individuals investigate themselves by making identity-defining commitments, which usually end by achieving a balance between personal interests and the vocational and ideological opportunities provided by surrounding context.

A turning point in identity development is the acquisition of personal autonomy. Scholars define autonomy as a multidimensional variable related to a set of phenomena involved in psychosocial development: the separation-individuation task as reported by Erikson (1968), management of detachment, and independence from family in order to look for new developmental environments, psychosocial maturity, self-regulation, self-control, self-efficacy, self-determination, and decision making (Noom et al., 2001).

The main theories on autonomy acquisition during adolescence stress the relation between the desire for autonomy and the development of beliefs about personal capabilities, the need to explore one’s own life goals and reflect on personal desires and preferences. As teenagers gain self-confidence and focus on personal goals and attitudes related to their individual interests and talents, the demand for autonomy in the household increases (Van Petegem et al., 2013). At the same time, intimate relationships with peers in adolescence acquire a vital importance for the definition of autonomous and personal identity. Adolescent friendships represent the possibility of strengthening the completion of the process of identification through establishing relationships with significant others (Jones et al., 2014).

As a privileged context of peer interaction and acquisition of knowledge and personal maturity, school greatly contributes to the development of adolescent identity and interpersonal relationships (Lannegrand-Willems and Bosma, 2006). Both curricular and extracurricular activities at school promote interpersonal interactions, and adolescents’ participation in school activities may have a protective role for academic achievement, substance use, sexual activity, psychological adjustment, delinquency, and young adult outcomes (Feldman and Matjasko, 2005).

During the COVID-19 emergency and the consequent lockdown in Italy, adolescents experienced a strong change in their personal and social environment, which could have affected the trajectory of their developmental tasks. Nevertheless, currently, there is a lack of knowledge of adolescents’ experience of living with COVID-19 and the main psychological issues related to it. Lockdown and the consequent closing of schools ushered in a new life routine for adolescents, centered on sharing time with family and temporarily interrupting face-to-face peer relationships. In this sense, similar to others, very impacting autobiographical events such as diseases or natural disasters, lockdown, and pandemic might have caused a biographical disruption (Bury, 1982; Tuohy and Stephens, 2012) interrupting developmental tasks typical of adolescence or forcing a reorganization. To understand the subjective experience of Italian adolescents and the potential impact of the biographical disruption on developmental tasks, we asked them the most impacting experiences related to COVID-19 and the national lockdown. We therefore collected narratives of positive and negative autobiographical events. Our main hypothesis was that the imposed lockdown may have constituted a turning point of pivotal developmental processes of autonomy acquisition and identity development, forcing adolescents to re-organize their personal resources. Therefore, we aimed to explore how Italian adolescents dealt with this peculiar life experience in terms of managing their developmental tasks.

Considering the lack of knowledge in literature and the need to investigate an unexplored topic, we performed a qualitative study to explore adolescents’ feelings and thoughts by means of their narratives. Qualitative research design helps “to generate useful knowledge about health and illness, from individual perceptions to how global systems work” (Green and Thorogood, 2018, p. 6) allowing for deep knowledge. Furthermore, narrative is a recognized tool to explore autobiographical experiences in terms of thoughts, emotions, and feelings as well as an intervention to promote emotional elaboration and meaning making (Pennebaker, 1997; Pennebaker et al., 2003). As a natural act to elaborate life episodes and generate meanings (Bruner, 1990), narrative enriches the search for evidence on autobiographical experience especially in both normative and not normative life transitions, when the need for meaning making about the self is strong. For this reason, the research design was exploratory, and it was caused by the need to generate insights on adolescence and COVID-19 starting from the direct adolescents’ narrated experience.

## Materials and Methods

### Participants

Participants of the present study were part of a broader study involving 5,295 Italian adolescents (mean age = 16.67, *SD* = 1.43; females = 75.2%; Min = 14, Max = 20) exploring emotional and cognitive patterns involved in COVID-19 experience. Since 14 is the Italian minimum age to give individual consent to having one’s online data processed, inclusion criteria for the present study were to be high school students and to be aged between 14 and 20. From the whole sample, we did not include data about adolescents with any missing data on either narrative task.

The final sample of 2,758 adolescents (females = 74.8%; mean age = 16.64, *SD* = 1.43; min = 14, max = 20; 14 years old = 7%, 15 years old = 17%, 16 years old = 22.4%, 17 years old = 22.2%, 18 years old = 23.2%, 19 years old = 6.3%, 20 years old = 1.9%) was composed by students attending lyceums (76.9%), technical high schools (16.9%), and vocational high schools (5.5%). Participants came from all regions of Italy: considering the impact of COVID-19 spread in Italy during data collection, the 16.8% of participants came from Lombardy (the most impacted region), the 20.7% came from medium impacted regions (Emilia Romagna, Liguria, Marche, Piedmont, Trentino Alto-Adige, Valle d’Aosta, Veneto), and the 62.5% came from other Italian region less impacted. Overall, 2,464 of them reported and narrated their most negative experiences and 2,110 reported their most positive experiences.

We also collected data about personal experiences involving COVID-19. Of the sample, 7.8% experienced a COVID-19 infection within the family circle (e.g., parents, brothers/sisters, grandparents, etc.). Of the sample, 38.6% experienced COVID-19 infections within friendship, scholastic, or broader social circles (e.g., neighbors, acquaintances). Ten participants (0.4%) reported to be infected themselves.

### Procedures and Data Analyses

After the approval of the ethical committee of the University of Florence, data collection took place from April 1 to April 5, 2020, during the peak of the COVID-19 outbreak in Italy, through a popular student website for sharing notes and receiving help with homework^1^, via a pop-up window asking the users to take part in the study. All respondents provided explicit informed consent at the beginning of the survey. It was possible to leave the survey at any point by simply closing the pop-up window. All data collected were anonymous. The contacts of a national helpline (i.e., telephone number and website chat) were provided at the end of the survey, inviting participants to get in touch if they need psychological support.

We invited participants to fill in two narrative tasks: the first on their most negative experience (“*Please, think about your memories surrounding COVID-19 and the “quarantine”. Would you please tell us your most negative experience during the last two weeks? Take your time and narrate what happened and how you experienced it. There are no limits of time and space for your narrative”*) and a second about their most positive experience of life during COVID-19 pandemic (“*Referring again to your memories surrounding COVID-19 and the “quarantine”, would you please tell us your most positive experience of the last two weeks? Please, narrate what happened and how you experienced that episode. There are no limits of time and space for your narrative”*).

The time frame of 2 weeks was referred to time approximately spent between the beginning of lockdown (March 9) and data collection.

All narratives were joined in two different full texts, one for the positive experience narratives and one for the negative ones. Then, a modeling emergent themes analysis was run by the T-Lab Software (Lancia, 2004). Modeling of Emergent Themes discovers, examines, and extrapolates the main themes (or topics) emerging from the text by means of co-occurrence patterns of key-term analysis by a probabilistic model, which uses the Latent Dirichlet Allocation (Blei et al., 2003). The results of the data analysis are several themes describing the main contents of a textual corpus. Researchers discussed in groups emergent themes and selected from elementary contexts derived from analysis those better explaining each theme.

This kind of textual analysis is therefore suggested in studies aiming to deepen unexplored topics in order to identify variables related to a specific kind of experience to be further investigated upon (Cortini and Tria, 2014).

## Results

First, the total word count of both narratives by the participants were analyzed. Adolescents’ negative experience narratives were composed of 76,007 words, with a mean number of 30.84 words per narrative, while 38,452 was the number of words used to narrate the most positive experiences (with a mean of 18.22 words per narrative collected). Table 1 shows the words mostly reported in the two texts.

**TABLE 1:** The occurrence of the most reported 20 words both for positive and negative experience narratives.

Negative experience narratives		Positive experience narratives	
		
Word	Occurrence	Word	Occurrence
Home (Casa)*	546	Time (Tempo)	435
**To see (Vedere)**	494	**Family (Famiglia)**	382
Negative (Negativo)	423	**Friend (Amico)**	296
**Friend (Amico)**	393	Positive (Positivo)	282
**Experience (Esperienza)**	345	**To feel (Sentire)**	241
**To feel (Sentire)**	340	**Home (Casa)**	212
**Person (Persona)**	310	**To see (Vedere)**	188
To exit (Uscire)	303	**Experience (Esperienza)**	179
Grandfather (Nonno)	284	To spend (Passare)	155
To live (Vivere)	263	**Moment (Momento)**	147
**Moment (Momento)**	249	**Person (Persona)**	139
Virus (Virus)	236	Beautiful (Bello)	124
To think (Pensare)	226	**School (Scuola)**	120
Fear (Paura)	221	Before (Prima)	113
**Day (Giorno)**	197	To manage (Riuscire)	91
Situation (Situazione)	190	**Day (Giorno)**	87
**School (Scuola)**	187	**Boyfriend (Ragazzo)**	81
**Family (Famiglia)**	170	Parent (Genitore)	80
**Boyfriend (Ragazzo)**	166	Happy (Felice)	75
Dear (Caro)	157	To cook (Cucinare)	71

*Shared words are highlighted in bold.

Looking at word occurrence in the two texts (positive and negative experience), many communalities emerged. Among the 20 most cited words in both texts there are: “Home,” “To See,” “Experience,” “Friend,” “To feel,” “Moment,” “Person,” “School,” “Day,” “Boyfriend,” and “Family.” Overall, 11 words out of 20 are shared between the vocabulary of the two collected narratives.

Looking at the modeling emergent themes analysis, The T-Lab software revealed four themes for each text. Tables 2, 3 summarize the emergent themes and the main words associated with each of them.

**TABLE 2:** Themes of negative experience narratives and main words for each of them.

Negative experience related to Covid-19											

Anguish and loss (34%)			Home as a limitation to autonomy (24%)			A new life routine (24%)			School as educational but not relational environment (18%)		
			
Key word (lemma)	Word frequency	Word total use	Key word (lemma)	Word frequency	Word total use	Key word (lemma)	Word frequency	Word total use	Key word (lemma)	Word frequency	Word total use
Grandfather (Nonno)	230	284	Year (Anno)	46	46	To stay (Restare)	37	45	Homeworks (Compiti)	84	93
Covid-19 (Covid-19)	105	105	To wait (Aspettare)	23	28	To respect (Rispettare)	28	32	Nervous (Nervoso)	30	30
To contract (Contrarre)	75	75	To ask (Chiedere)	21	21	Body (Fisico)	21	21	Lesson (Lezione)	30	40
Uncle (Zio)	75	85	To write (Scrivere)	19	19	Rules (Regole)	20	20	To distress (Angosciare)	28	28
Hospital (Ospedale)	75	96	Pleasure (Piacere)	17	20	Decree (Decreto)	20	22	Study (Studio)	27	36
To discover (Scoprire)	70	82	To begin (Cominciare)	17	22	Aware (Consapevole)	20	23	On-line (On-line)	23	23
Mum (Mamma)	52	65	Minute (Minuto)	15	15	To face (Affrontare)	18	21	TV (TV)	20	20
Neighbor (Vicino)	50	67	Relationship (Rapporto)	15	15	To get used to (Abituare)	16	21	First (Primo)	19	19
Risk (Rischio)	37	37	Sense (Senso)	15	15	To eat (Mangiare)	16	16	Severity (Gravità)	19	19
To infect (Contagiare)	37	47	Bedroom (Camera)	14	14	Sensations (Sensazioni)	15	15	Pictures (Immagini)	19	19
Health (salute)	37	47	To endure (Sopportare)	14	14	Dog (Cane)	14	14	Military (Militare)	18	18
Sister (Sorella)	35	43	To stop (Smettere)	13	13	Breathing mask (Mascherina)	14	14	Bergamo (Bergamo)	18	19
Brother (Fratello)	32	38	Sun (Sole)	12	12	Daily (Quotidianamente)	12	12	Inquiry (Interrogazione)	16	16
Cousin (Cugino)	30	32	Hope (Speranza)	12	12	Filter (Guanto)	12	12	To tell (Raccontare)	16	16
To be afraid (Temere)	28	35	Tear (Lacrima)	12	12	Memories (Ricordi)	12	13	Newscast (Telegiornale)	15	15
Elder (Anziano)	27	28	High School (Liceo)	12	12	Emotional (Emotivo)	11	11	Therapy (Terapia)	15	17
Severe (Grave)	27	34	Common (Comune)	11	11	Embrace (Abbraccio)	10	10	Test (Verifica)	13	13
To leave (Partire)	21	21	To exit (Uscire)	11	11	Governament (Governo)	10	10	Dejection (Sconforto)	10	10
Concern (Preoccupazione)	20	25	Hate (Odio)	10	11	War (Guerra)	9	9	Rabbia (Anger)	10	11
Region (Regione)	19	25	Nervousness (Nervosismo)	9	9	Collapse (crollo)	9	9	To organize (organizzare)	9	9
Milan (Milano)	19	21	To provoke (Provocare)	9	9	Desktop (Schermo)	9	10	Uncertainty (Incertezza)	9	10
Funeral (Funerale)	18	18	To drop (Abbandonare)	9	9	Activity (Attività)	9	11	To reassure (Tranquillizzare)	9	10
Disease (Disease)	14	14	To dilate (dilatare)	8	8				To terrorize (Terrorizzare)	9	10
Ward (Reparto)	13	13	To wake up (Svegliarsi)	8	8						
North (Nord)	12	12									
Shocked (Turbato)	10	10	To stress (Stressare)	8	9						
Epidemy (Epidemia)	10	13									
Depressed (Depresso)	9	10									

*The saturation percentage of words explained by each theme has been reported.*

*The table shows words with a degree of association within every theme ≥ 0.75.*

*Lemmas are reported following their occurrence in the tale.*

**TABLE 3:** Themes of positive experience narratives and specific words for each of them.

Positive experience related to COVID-19											

Discovering oneself (33%)			Sharing the life at distance (31%)			Re-discovering the family (22%)			To be part of an extraordinary experience (14%)		
			
Key word (lemma)	Word frequency	Word total use	Key word (lemma)	Word frequency	Word total use	Key word (lemma)	Word frequency	Word total use	Key word (lemma)	Word frequency	Word total use
To start (Iniziare)	65	65	Friend (Amico)	225	296	Family (Famiglia)	299	382	Week (Settimana)	43	56
To understand (Capire)	55	62	Videocall (Videochiamata)	66	66	To play (Giocare)	57	69	Unique (Unico)	40	52
To catch (Prendere)	51	67	Birthday (Compleanno)	48	52	Mum (Mamma)	49	55	Last (Ultimo)	22	24
Small (Piccolo)	30	32	Together (Insieme)	43	43	Step (Passo)	35	35	Particular (Particolare)	17	17
Re-start (Ricominciare)	26	26	Video (Video)	43	47	Re-discovery (Riscoprire)	35	40	Fear (Paura)	15	20
TV Series (Serie TV)	24	25	To call (Chiamare)	41	41	Fratello (Brother)	25	25	To Lose (Perdere)	12	12
Music (Musica)	24	25	To see (Vedere)	29	29	Games (Giochi)	19	19	Experience (Esperienza)	10	10
Homeworks (compiti)	23	23	Videochat (Videochat)	29	37	To join (Unirsi)	16	22	Test (Verifica)	10	10
To play (Suonare)	22	22	Classmates (Compagni)	27	35	To spend (Trascorrere)	15	20	Routine (Routine)	9	9
To concentrate on (Concentrarsi)	20	22	Town (Paese)	20	24	Society (Società)	13	16	First (Primo)	8	9
Balcony (Balcone)	20	20	To celebrate (Celebrare)	19	19	To stay (Restare)	13	17	Number (Numero)	8	9
Importance (Importanza)	20	24	Far (Lontano)	17	17	To work-out (Allenarsi)	12	14	Inquiry (Interrogazione)	8	9
Exercizes (Esercizi)	18	19	Evening (Sera)	17	17	To reconnect (Riallacciare)	12	14	To be part of (Partecipare)	8	12
Lack (Mancanza)	17	17	To love (Amare)	14	14	Paper (Carta)	12	16	To play (Giocare)	7	9
To leave (Lasciare)	117	17	Shopping (Spesa)	13	15	Value (Valore)	11	16			
To Appreciate (Apprezzare)	20	24	Joy (Gioia)	11	11	Netflix (Netflix)	8	9			
World (Mondo)	15	15	Chat (Chat)	11	11	Dinner (Cena)	8	9			
To hope (Sperare)	15	15									
To enjoy (Divertirsi)	10	11	Saturday (Sabato)	10	10						
To paint (dipingere)	13	14									
To become (Diventare)	13	14	Boyfriend (Fidanzato)	10	10						
Care (Cura)	12	12									
To Listen (Ascoltare)	11	11	Proximity (Vicinanza)	9	9						
			Affection (Affetto)	9	9						

*The saturation percentage of words explained by each theme has been reported.*

*The table shows words with a degree of association within every theme ≥ 0.75.*

*Lemmas are reported following their occurrence in the tale.*

### Negative Experiences Narratives

For negative experiences narratives, the first and most representative theme concerned feelings of “Anguish and Loss” and was explained by 34% of lemmas. Adolescents shared their anguish for having lost physical and emotional contact with relatives due to quarantine: *“I went to my grandmother’s house because she lives next door. I went to hug her and she pushed me away as if I stank, it was so ugly, I felt like a stranger” (Participant n. 1070, male 17 years old)*. In this narrative, social distancing acquired the meaning of loss of intimacy in close relationships; other adolescents narrated their anguish for their parents’ and relatives’ health due to the spread of COVID-19. One participant wrote: *“When I heard that both my parents were going to have to go back to work, I got very scared, and I’m still scared for their health. We have a lot of friends who are sick, some are dead and we couldn’t even say goodbye to them” (Participant n. 1234, male 16 years old).*

The inability to say goodbye to relatives and friends and to experience contact with their deaths is a frequent issue in collected narratives. As shared by a female participant, grief is a process hindered by the inability to experience loss directly: *“The most negative experience I had was the death of my grandfather, who died after contracting COVID-19. You will think that I’m only talking about the loss itself, but actually difficulties came later. Not because there were people crying at the funeral and I had to show myself strong in front of my parents; not because when I went to his house I couldn’t find him; not because I won’t get to be spoiled by him just like every other granddaughter is by her grandfather; but because I had to undergo this process with just my mind. I had to imagine a funeral, I had to imagine him, pale and cold, in the coffin and try to feel the dampness of the tears on my cheeks at the moment of burial. There was nothing to help me metabolize the death, to make it happen in my mind. I’m usually a crybaby, but when they told me that my grandfather died I cried only once. When I think about it I feel guilty for how insensitive I’ve been, but he’s still there for me, when I think of him I see him alive. I tried to kill him with my thoughts because that’s the reality, but how hard is it to understand someone’s death when you don’t face it? When you don’t live it?” (Participant n. 23, female 16 years old)*.

The second theme explained 24% of lemmas and it was labeled “Home as a limitation to autonomy.” Participants narrated their experience of feeling a limitation to their personal autonomy in daily life activities.

A female participant narrated: *“Staying at home brings me moments of nervousness and I’m easily irritable. I often have panic attacks, precisely because staying at home for so long is not good for me. One feels alone, like in a cage and suffocated feelings give rise to nervousness that causes tension” (Participant n. 645, female 16 years old).* Similarly, the following narrative introduces the difficulty of finding a personal space to give voice to individual needs at home: *“It’s very hard for me to concentrate and I can’t stand spending 24 hours a day with my parents arguing. I don’t even have my own bedroom, because the door is missing so I have to be with them all the time. Personally, I’m not afraid of the virus, there have always been cases in history and of course we have always come out of it unscathed; the point is that I just want to go back to having the chance to be away from home, for example at school and possibly soon at university” (Participant n. 2185, female 18 years old)*. The two negative experiences suggested adolescents’ perception of living with COVID-19 as a time to forcibly lose their personal autonomy.

Another male adolescent shared the sensation of being in prison as the result of having lost an individual identity related to a state of suspension of personal desires and identity: *“It’s bad to wake up in the morning knowing you can’t accomplish anything with your life, you can’t do anything. I look out the window and it’s all deserted, no more sounds of cars, buses or people talking. It’s like a changed world, it’s like being in prison for something that’s not your fault. All I can do is wait and stay at home.”* (Participant n. 1460, 16 years old).

The third theme, saturating by 24% of lemmas, concerned the impact of “A new life routine.” Adolescents narrated their contact with life in quarantine as well as social distancing. Participant n. 488, a 17-year-old male, narrated his most negative experience of not recognizing his best friend because of the mask: *“The worst experience I had was when I went out for the first time to go shopping, wearing a mask and gloves. It was horrible to see used masks and gloves in the street that someone threw on the ground. Across the street someone said goodbye to me. He was my best friend with his dog, but I didn’t recognize him because he was covered by the mask. My best friend!”* Narratives reported the adolescents’ difficult impact with a new daily routine in which their closest relationships (best friend) and daily activities (shopping) acquire the meaning of something unusual and perturbing. Similarly, the following extract focused on the feeling of being aware of taking part in a new life routine, which is completely different from one’s wishes about adolescent life: *“There is not one episode in particular, but perhaps there is the most negative ‘feeling’ of this period, and it is certainly awareness. It’s being aware that you can’t live your senior year in high school as you would have liked. It’s the awareness of not being able to kiss your mom who just came back from the supermarket with your favorite dessert. It’s the awareness that you can’t go dancing or simply talk with friends about something that isn’t the ‘war bulletin’ or the press conference that resounds in the homes of Italians every night at 6 p.m.” (Participant n. 359, female 16 years old).*

The fourth emergent theme was saturated by 18% of lemmas and was labeled “School as educational but not relational environment.” Participants reported the difficulty of being engaged in educational activities, which are perceived as lacking in social opportunities. A male adolescent (n. 60, 17 years old) reported: *“since there have been positive cases I’ve stayed at home, but with the online lessons and lots of homework I am getting sad and especially stressed. I wanted to talk about Bergamo with the teachers and my classmates, but there is no time and in the online lessons we only talk about school and homework.”*

A participant expressed his feeling of being unwelcome and misunderstood by teachers due to the relational distance: *“In my opinion this is the saddest thing that this virus has brought: we young people no longer believe in dreams, but above all in hope for a better future. The professors, instead of understanding this situation, blame us, saying that we are ‘slackers’ and that we think we are on holiday, punishing us with millions of tasks, depriving us of everything. […] So, these are the reasons why we young people are exhausted and full of repressed hatred, because we see our peers die before our eyes and teachers often don’t understand us” (Participant n. 2545, female 17 years old).*

Moreover, homework and online classes work as stressors and increase the lack of relations: *“I felt agitated because homework and video tutorials have stressed me so much. It’s not the same online. I understand the gravity of the situation, the images we see are terrible, all those coffins. I miss my class, the teacher coming in, everything” (Participant n. 260, male 17 years old).*

### Positive Experiences Narratives

Concerning positive experiences, four themes emerged from the modeling analysis.

The first theme, the most representative for positive experiences collected, covered 33% of the lemmas and dealt with “Discovering oneself.” Adolescents reported to have discovered the pleasure of spending time with themselves and dedicating time to reading, listening to music, painting, and working out on their own. In this sense, lockdown became an opportunity for self-disclosure and personal growth: *“I read, studied, I’ve cooked various stuff, experimented, relaxed taking time for myself, watched TV series, movies, played chess. Everything that made me feel good. I felt accepted by myself, because I had time to think about myself much more and to reflect, making me feel like a better and acceptable person”* (Participant n. 2069, male 15 years old).

Similarly, a girl narrated: *“Like never before, I have time to look inside and talk to myself in my bedroom, having more doubts, being able to resolve them, or simply leaving them unresolved, discovering what confuses me and understanding who I am” (Participant n. 1369, female 18 years old)*.

The second emergent theme was labeled “Sharing life at a distance” (31% of lemmas) and dealt with the opportunity to be in a close relationship even at a distance. A participant narrated his relief in feeling his best friend’s support via video-call: *“I Hear my friend tell me on the video-call that everything’s going to be okay and we’re going to come out of this even stronger. She said, ‘We’ll come back and watch the sunset on the beach, we’ll come back and eat ice cream together, we’ll come back and hug everybody, have faith’. I felt safe and full of hope” (Participant n. 2721, male 14 years old).*

Friendship as an anchor is a frequent issue in adolescents’ narratives: *“I felt a big panic inside and I had a video-call with all my friends at 1 am in a tense moment, it helped me a lot!” (Participant n. 1970, female 17 years old).*

The third emerged theme, named “Re-discovering family,” was saturated by 22% of the lemmas and focused on the positive impact of spending time with family members and discovering the joy of doing things together: *“I’m realizing how precious time is, every moment must be enjoyed because we could be deprived of it at any moment. I spend more time with my parents, before they were always at work and I used to see them for a few hours” (Participant n. 881, female, 16 years old).* Similarly, a boy narrated the positive value of spending time with his grandparents: *“I’m spending a lot of time with my grandparents and I’m growing up because they teach me so many things I didn’t know! We’ve rediscovered board games and we often play them all together” (Participant n. 2648, 17 years old).*

The last theme, “To be part of an extraordinary experience”, was saturated by 14% of the lemmas and concerned participants’ feeling of being part of an unusual experience, which will have an impact on the culture they are living in. A participant narrated: *“When I’m in class and I see my classmates, even if we do a test or an inquiry, it’s still a unique experience that I will tell my kids about!” (Participant n. 2044, male 18 years old).* Most of the participants reported their satisfaction in their perception of having an active role in society by following the rules of social distancing and protecting others from contagion: *“For once I really felt like a fundamental part of society” (Participant 1841, female 15 years old)*.

## Discussion

The present study aimed to explore adolescents’ experience of living during the COVID-19 emergency and national lockdown in terms of narratives on positive and negative experiences. In light of a lack of scientific evidence on adolescents’ experience of living with infectious diseases and under national lockdown, the present study brings knowledge on negative and positive issues of such an impactful experience in this peculiar developmental age of adolescence.

At first, results show that adolescents were more forthcoming about their negative experiences than about positive ones. This datum is not a surprise: scientific literature defines one’s need to “create coherence out of chaos” (Fivush et al., 2003, p. 1). Scientific literature highlights that negative narratives are usually longer and more coherent than positive ones, and this is due to the narrator’s need to elaborate autobiographical past by means of language (Fioretti and Smorti, 2015, 2017).

Looking at word occurrence in both texts, results show similarities between terms used to describe the most negative and positive experiences. Nevertheless, emergent themes put in light different issues related to the same words. Overall, results highlight indeed a complex experience of adolescents characterized by a developmental challenge that may entail risk factors, as in the case of loss and anguish related to illness and contagion, or protective factors, such as the possibility of transforming the COVID-19 experience into an opportunity for personal growth.

In the case of impacting experiences such as diseases or traumatic events, scholars introduced the construct of biographical disruption (Bury, 1982; Fioretti and Smorti, 2014), which determines a strong breakdown in one’s life trajectory forcing the individual to restore it finding a continuity between past, present, and future. Concerning COVID-19, our results point out that such a biographical disruption may be associated with the interruption of important developmental tasks such as personal autonomy (Alonso-Stuyck et al., 2018). Of the adolescents’ lemmas, 24% narrated lockdown as a stressor in their process of constructing an individual physical and mental environment separate from the family one.

As shown by narratives on positive experiences of living with COVID-19, home acquires a duplex meaning in adolescents’ lives: loss of autonomy, but also the place where re-discovering family as a protective factor thanks to the opportunity to share activities and to spend time together. As argued by Guessoum et al. (2020), family time is related with less depression symptoms in adolescents. Moreover, our results suggest that family can play an active role in the co-construction of what it means to live during a pandemic and can provide support during experiences of loss, which, as results show, appear to be the most represented issue in adolescents’ narratives.

As reported by participants, the impossibility of experiencing a direct contact with loss and death may play a traumatic role in adolescents’ lives. In their narratives, grief is forcibly an intimate and individual process in which, as in the case of traumatic events, the disruption is sudden and unexpected. Starting from these results, further investigation on potential posttraumatic disorders and long-term symptoms in adolescents related to COVID-19 is needed.

If family plays a protective role in collected narratives, adolescents denounce the absence of school as a place for relationships and emotional sharing. Participants narrate how they feel like receptors of educational contents without being able to play an active role within the educational process. Passivity and the inability to find a space to share concerns and emotions about the impact of the COVID-19 disease on their lives are the base of a feeling of disconnection from the educational environment. In this sense, the current “absence” of school may constitute a risk factor in adolescents’ development, as described in scientific literature (Feldman and Matjasko, 2005).

School closing is part of a broader spectrum of the breakdown of the daily routine that participants described as a negative experience. In developmental psychology, routines acquire a pivotal role in fostering the security necessary for the process of autonomy and self-definition, in childhood and adolescence (Crocetti, 2018). In this sense, the new life routine of wearing masks and gloves, and performing social distancing strongly impacts the process of creating one’s own identity.

On the other hand, narratives on positive experiences also see COVID-19 as an opportunity to make contact and define certain aspects of one’s identity that have not yet been considered. As shown, the discovery of oneself plays a pivotal role in positive experiences narratives saturating 33% of lemmas in analysis.

Identity, as described by Marcia et al. (2012), undergoes a strong process of moratorium which, as results suggest, during the quarantine also becomes a path of deeper research into one’s sense of self, without the pressure of external agents. The discovery of the self-emergent theme suggests the hypothesis of a posttraumatic growth (PTG) related to life during the COVID-19 emergency. Participants narrated their individual research of themselves and the discovery of the importance of intimate reflexivity. In literature, over time, several terms have been used to describe the positive changes experienced by a person as a result of stress: “perceived benefits” (Calhoun and Tedeschi, 1991), “raising existential awareness” (Yalom and Lieberman, 1991), “stress-related growth” (Park et al., 1996), and “growth through adversity” (Joseph and Linley, 2006). Posttraumatic growth has been defined as an individual transformation entailing both positive intrapersonal and interpersonal changes caused by the impact of facing life challenges (Tedeschi and Calhoun, 1995). Our results suggest that, together with the importance of sharing experiences with peers as reported in 31% of lemmas about positive experiences, an intimate developmental process of self-moratorium was facilitated by living in lockdown due to the COVID-19 emergency. Adolescents narrate their discovery of alone-time as a personal process of growth. Studies on PTG during adolescence are still poor (Milam et al., 2004) and suggest the importance of investigating potential specificities of growth in this peculiar developmental age and its correlations. Future studies could explore the construct of PTG in adolescents exposed to the COVID-19 pandemic in order to further assess a positive impact of living with the current emergency in their lives.

## Limitations and Conclusion

Although it provides evidence on a topic which is unknown, the present study has some limitations. First, we did not control for narrative task administration order. All participants completed first the narrative on negative memories and, second, the one on positive experiences. For this reason, the present study did not aim to compare negative and positive experience, rather it considers them as separate narratives on autobiographical experience of living with COVID-19 pandemic.

Moreover, the sample is composed of a large percentage of females and of high school students and does not consider the portions of adolescents of the Italian population who are not currently involved in education. Further studies should consider adolescents’ varying economic and cultural backgrounds. A second limitation is related to the varying impact of the COVID-19 emergency in the different regions of Italy. Adolescents’ experiences might be related to having or not having personal contacts with the disease in their family or social environment. Future studies should focus on specific developmental challenges due to direct or indirect contact with COVID-19.

A third limitation is related to the lack of consideration of the interindividual differences. The study describes a process related to the COVID-19 in the global population without considering possible differential impacts related to personal characteristics and vulnerabilities.

To conclude, the results suggest the need to take into account the impact of lockdown in the developmental tasks of adolescence. As for the negative experiences, loss of autonomy and anguish related to death and loss are the most representative topics. Further studies could better investigate the autonomy issue related to COVID-19 emergency considering the role family and different parenting models can play. For instance, very few studies have investigated the role of pre-pandemic maltreatment experience (Guo et al., 2020) or other experience related to family environment. Our results suggest the duplex role of family and invite scholars and professionals to design specific intervention programs for adolescents with family vulnerability.

Conversely, school, a pivotal developmental environment according to scientific literature, represented a smaller percentage of words in the narratives we collected for our sample, suggesting the need to debate on the lack of relation adolescents perceive in online didactic activities. Home and family may play a double role, both limiting adolescents’ acquisition of autonomy and providing an enriching setting for their personal growth. The latter, discovering oneself, is the most representative in positive experience narratives. In this sense, the starting hypothesis of the present study was left partially unconfirmed. Lockdown and life during the COVID-19 emergency may activate both a disruption and an empowering process in adolescents’ developmental tasks. Further studies are needed on psychological and social variables promoting or contrasting both processes.

In the light of scarce studies exploring narratives on COVID-19 experience, the present research supports the importance of giving language to the autobiographical past by means of methods exploring qualitatively participants’ experience. Results show that a narrative is a tool to collect information on personal experience and to generate insight starting from it. Additionally, a narrative allows narrators emotional disclosure and to give meaning to their life story (Bruner, 1990; McAdams et al., 2006). This meaning-making process is even more important in developmental ages, as adolescence is, characterized by self and identity definition and growth of autobiographical process skills (Habermas and Bluck, 2000). We support the need to further investigate adolescents’ narratives in this pandemic transition both as a tool to collect data and as an intervention to promote well-being through emotional and intrapsychic disclosure.

## Data Availability Statement

The raw data supporting the conclusions of this article will be made available by the authors, without undue reservation, to any qualified researcher.

## Ethics Statement

The studies involving human participants were reviewed and approved by the Commissione per l’Etica della Ricerca, Università degli Studi di Firenze. Written informed consent to participate in this study was provided by the participants’ legal guardian/next of kin.

## Author Contributions

CF, BP, AN, and EM conceived and performed the study design, data collection, and mastered the data. CF ran the data analysis. BP, AN, and EM discussed the results. CF wrote the manuscript with the support of BP and AN. EM and AN supervised the project and manuscript preparation. All authors contributed to the article and approved the submitted version.

## Conflict of Interest

The authors declare that the research was conducted in the absence of any commercial or financial relationships that could be construed as a potential conflict of interest.

## Footnotes

1 www.skuola.net

## References

[B1] Alonso-Stuyck P. Zacarés J. J. Ferreres A. (2018). Emotional separation, autonomy in decision-making, and psychosocial adjustment in adolescence: a proposed typology. *J. Child Fam. Stud.* 27 1373–1383. 10.1007/s10826-017-0980-5

[B2] Blei D. M. Ng A. Y. Jordan M. I. (2003). Latent dirichlet allocation. *J. Mach. Learn. Res.* 3 993–1022.

[B3] Bruner J. S. (1990). *Acts of Meaning.* Cambridge, MA: Harvard university press.

[B4] Bury M. (1982). Chronic illness as biographical disruption. *Sociol. Health Illness* 4 167–182. 10.1111/1467-9566.ep11339939 10260456

[B5] Calhoun L. G. Tedeschi R. G. (1991). Perceiving benefits in trau matic events: some issues for practicing psychologists. *J. Train. Pract. Prof. Psychol.* 5 45–52.

[B6] Cortini M. Tria S. (2014). Triangulating qualitative and quantitative approaches for the analysis of textual materials: an introduction to T-lab. *Soc. Sci. Comput. Rev.* 32 561–568. 10.1177/0894439313510108

[B7] Crocetti E. (2018). Identity dynamics in adolescence: processes, antecedents, and consequences. *Eur. J. Dev. Psychol.* 15 11–23. 10.1080/17405629.2017.1405578

[B8] Dodgson J. E. Tarrant M. Chee Y.-O. Watkins A. (2010). New mothers’ experiences of social disruption and isolation during the severe acute respiratory syndrome outbreak in Hong Kong. *Nurs. Health Sci.* 12 198–204. 10.1111/j.1442-2018.2010.00520.x 20602692

[B9] Ellis W. E. Dumas T. M. Forbes L. M. (2020). Physically isolated but socially connected: Psychological adjustment and stress among adolescents during the initial COVID-19 crisis. *Can. J. Behav.* 52 177–187. 10.1037/cbs0000215

[B10] Erikson E. H. (1968). *Identity Youth and Crisis.* New York, NY: W.W. Norton Inc.

[B11] Feldman A. F. Matjasko J. L. (2005). The role of school-based extracurricular activities in adolescent development: a comprehensive review and future directions. *Rev. Educ. Res.* 75 159–210. 10.3102/00346543075002159

[B12] Fioretti C. Smorti A. (2014). Improving doctor-patient communication through an autobiographical narrative theory. *Commun. Med.* 11 275–284. 10.1558/cam.v11i3.20369

[B13] Fioretti C. Smorti A. (2015). How emotional content of memories changes in narrative. *Narrat. Inq.* 25 37–56. 10.1075/ni.25.1.03fio

[B14] Fioretti C. Smorti A. (2017). Narrating positive versus negative memories of illness: does narrating influence the availability and the emotional involvement of memories of illness?. *Eur. J. Cancer Care* 23 1–7. 10.1111/ECC.12524 27271542

[B15] Fivush R. Hazzard A. McDermott Sales J. Sarfati D. Brown T. (2003). Creating coherence out of chaos? Children’s narratives of emotionally positive and negative events. *Appl. Cogn. Psychol.* 17 1–19. 10.1002/acp.854

[B16] Green J. Thorogood N. (2018). *Qualitative Methods for Health Research.* Thousand Oaks, CA: Sage Pubblications.

[B17] Guessoum S. B. Lachal J. Radjack R. Carretier E. Minassian S. Benoit L. (2020). Adolescent psychiatric disorders during the COVID-19 pandemic and lockdown. *Psychiatry Res.* 291:113264. 10.1016/j.psychres.2020.113264 32622172 PMC7323662

[B18] Guo J. Fu M. Liu D. Zhan B. Wang X. van IJzendoorn M. H. (2020). Is the psychological impact of exposure to COVID-19 stronger in adolescents with pre-pandemic maltreatment experiences? a survey of rural chinese adolescents. *Child Abuse Neglect.* 2020:104667. 10.1016/j.chiabu.2020.104667 32859393 PMC7440157

[B19] Habermas T. Bluck S. (2000). Getting a life: the emergence of the life story in adolescence. *Psychol. Bull.* 126:748. 10.1037/0033-2909.126.5.748 10989622

[B20] Havighurst R. J. (1948). *Developmental Tasks and Education.* Chicago: University of Chicago Press.

[B21] Italian Ministry of Health (2020). *COVID-19 Daily New Cases and Deaths in Italy.* Rome: Italian Ministry of Health.

[B22] Johns Hopkins Coronavirus Resource Center (2020). *Cases and Mortality by Country.* Available online at: https://coronavirus.jhu.edu/ (accessed July 13, 2020).

[B23] Jones R. M. Vaterlaus J. M. Jackson M. A. Morrill T. B. (2014). Friendship characteristics, psychosocial development, and adolescent identity formation. *Pers. Relationsh.* 21 51–67. 10.1111/pere.12017

[B24] Joseph S. Linley P. A. (2006). Growth following adversity: theoretical perspectives and implications for clinical practice. *Clin. Psychol. Rev.* 26 1041–1053. 10.1016/j.cpr.2005.12.006 16473442

[B25] Kroger J. Marcia J. E. (2011). “The identity statuses: origins, meanings, and interpretations,” in *Handbook of Identity Theory and Research*, eds Schwartz S. J. Luyckx K. Vignoles V. L. (New York, NY: Springer), 31–53. 10.1007/978-1-4419-7988-9_2

[B26] Kroger J. Martinussen M. Marcia J. E. (2010). Identity status change during adolescence and young adulthood: a meta-analysis. *J. Adolesc.* 33 683–698. 10.1016/j.adolescence.2009.11.002 20004962

[B27] Lancia F. (2004). *Strumenti Per L’analisi dei Testi [Tools for Textual Analysis].* Milan: FrancoAngeli.

[B28] Lannegrand-Willems L. Bosma H. A. (2006). Identity development-in-context: the school as an important context for identity development. *Identity* 6 85–113. 10.1207/s1532706xid0601_6 26627889

[B29] Lee J. (2020). Mental health effects of school closures during COVID-19. *Lancet Child Adolesc. Health* 4:421 10.1016/S2352-4642(20)30109-7 PMC7156240 32302537

[B30] Main A. Zhou Q. Ma Y. Luecken L. J. Liu X. (2011). Relations of SARS-related stressors and coping to Chinese college students’ psychological adjustment during the 2003 Beijing SARS epidemic. *J. Counsel. Psychol.* 58 410–423. 10.1037/a0023632 21574694

[B31] Marcia J. E. Waterman A. S. Matteson D. R. Archer S. L. Orlofsky J. L. (2012). *Ego Identity: A Handbook for Psychosocial Research.* Berlin: Springer Science & Business Media.

[B32] McAdams D. P. Bauer J. J. Sakaeda A. R. Anyidoho N. A. Machado M. A. Magrino-Failla K. (2006). Continuity and change in the life story: a longitudinal study of autobiographical memories in emerging adulthood. *J. Pers.* 74 1371–1400. 10.1111/j.1467-6494.2006.00412.x 16958706

[B33] Milam J. E. Ritt-Olson A. Unger J. B. (2004). Posttraumatic growth among adolescents. *J. Adolesc. Res.* 19 192–204.

[B34] Noom M. J. Deković M. Meeus W. (2001). Conceptual analysis and measurement of adolescent autonomy. *J. Youth Adolesc.* 30 577–595. 10.1023/a:1010400721676

[B35] Oosterhoff B. Palmer C. A. Wilson J. Shook N. (2020). Adolescents’ motivations to engage in social distancing during the COVID-19 pandemic: associations with mental and social health. *J. Adolesc. Health* 67 179–185. 10.1016/j.jadohealth.2020.05.004 32487491 PMC7205689

[B36] Park C. L. Cohen L. H. Murch R. L. (1996). Assessment and prediction of stress-related growth. *J. Pers.* 64 71–105. 10.1111/j.1467-6494.1996.tb00815.x 8656319

[B37] Peng E. Y.-C. Lee M.-B. Tsai S.-T. Yang C.-C. Morisky D. E. Tsai L.-T. (2010). Population-based post-crisis psychological distress: an example from the SARS outbreak in Taiwan. *J. Form. Med. Assoc.* 109 524–532. 10.1016/S0929-6646(10)60087-3 PMC3106105 20654792

[B38] Pennebaker J. W. (1997). *Opening Up: The Healing Power of Expressing Emotions.* New York, NY: Guilford Press.

[B39] Pennebaker J. W. Mehl M. R. Niederhoffer K. G. (2003). Psychological aspects of natural language use: our words, our selves. *Annu. Rev. Psychol.* 54 547–577. 10.1146/annurev.psych.54.101601.145041 12185209

[B40] Qiu J. Shen B. Zhao M. Wang Z. Xie B. Xu Y. (2020). A nationwide survey of psychological distress among Chinese people in the COVID-19 epidemic: implications and policy recommendations. *Gen. Psychiatr.* 33 1–3. 10.1136/gpsych-2020-100213 32215365 PMC7061893

[B41] Rajkumar R. P. (2020). COVID-19 and mental health: a review of the existing literature. *Asian J. Psychiatr.* 52:102066. 10.1016/j.ajp.2020.102066 32302935 PMC7151415

[B42] Rossi R. Socci V. Talevi D. Mensi S. Niolu C. Pacitti F. (2020). COVID-19 pandemic and lockdown measures impact on mental health among the general population in Italy. An N = 18147 web-based survey. *medRxiv* [Preprint], 10.1101/2020.04.09.20057802 PMC7426501 32848952

[B43] Tedeschi R. G. Calhoun L. G. (1995). *Trauma & Transformation: Growing in the Aftermath of Suffering.* Thousand Oaks, CA: Sage Publications Inc.

[B44] Tuohy R. Stephens C. (2012). Older adults’ narratives about a flood disaster: resilience, coherence, and personal identity. *J. Aging Stud.* 26 26–34. 10.1016/j.jaging.2011.06.002

[B45] Van Bortel T. Basnayake A. Wurie F. Jambai M. Koroma A. S. Muana A. T. (2016). Psychosocial effects of an Ebola outbreak at individual, community and international levels. *Bull. World Health Organ.* 94 210–214. 10.2471/BLT.15.158543 26966332 PMC4773931

[B46] Van Petegem S. Vansteenkiste M. Beyers W. (2013). The jinglejangle fallacy in adolescent autonomy in the family: in search of an underlying structure. *J. Youth Adolesc.* 42 994–1014. 10.1007/s10964-012-9847-7 23111845

[B47] Wang C. Pan R. Wan X. Tan Y. Xu L. Ho C. S. (2020a). Immediate psychological responses and associated factors during the initial stage of the 2019 Coronavirus disease (COVID-19) epidemic among the general population in China. *Intern. J. Environ. Res. Public Health* 17:1729. 10.3390/ijerph17051729 32155789 PMC7084952

[B48] Wang C. Pan R. Wan X. Tan Y. Xu L. McIntyre R. S. (2020b). A longitudinal study on the mental health of general population during the COVID-19 epidemic in China. *Brain Behav. Immun.* 87 40–48. 10.1016/j.bbi.2020.04.028 32298802 PMC7153528

[B49] Xie X. Xue Q. Zhou Y. Zhu K. Liu Q. Zhang J. (2020). Mental Health Status Among children in home confinement during the Coronavirus disease 2019 outbreak in Hubei province, China. *JAMA Pediatr.* 174 898–900. 10.1001/jamapediatrics.2020.1619 32329784 PMC7182958

[B50] Yalom I. D. Lieberman M. A. (1991). Bereavement and heightened existential awareness. *Psychiatry* 54 334–345. 10.1080/00332747.1991.11024563 1788364

[B51] Yeung D. Y.-L. Fung H. H. (2007). Age differences in coping and emotional responses toward SARS: a longitudinal study of Hong Kong Chinese. *Aging Ment. Health* 11 579–587. 10.1080/13607860601086355 17882596

